# A Phase 1b study of the OxPhos inhibitor ME-344 with bevacizumab in refractory metastatic colorectal cancer

**DOI:** 10.1007/s10637-024-01489-1

**Published:** 2024-12-27

**Authors:** Patrick M. Boland, Heinz-Josef Lenz, Kristen K. Ciombor, Vaia Florou, Michael J. Pishvaian, Michael Cusnir, Deirdre Cohen, Jessie Y. Guo, Min Tang, Prabhu Rajagopalan, Sandra E. Wiley, Richard G. Ghalie, Howard S. Hochster

**Affiliations:** 1 Division of Oncology, Rutgers Cancer Institute of New Jersey, New Brunswick, NJ USA; 2 Division of Medical Oncology, University of Southern California Norris Comprehensive Cancer Center, Los Angeles, CA USA; 3 Division of Hematology/Oncology, Vanderbilt University Medical Center, Nashville, TN USA; 4 Division of Oncology, Huntsman Cancer Institute of the University of Utah, Salt Lake City, UT USA; 5 Department of Medicine, Johns Hopkins Kimmel Cancer Center, Washington, DC USA; 6 Division of Hematology & Oncology, Mount Sinai Medical Center, Miami Beach, FL USA; 7 Department of Hematology Oncology, Icahn School of Medicine at Mount Sinai, New York, NY USA; 8 Statbeyond Consulting, Irvine, CA USA; 9 PR Consulting, Mountain Lakes, NJ USA; 10 MEI Pharma, San Diego, CA USA; 11 Rutgers Cancer Institute of New Jersey, 195 Little Albany St, New Brunswick, NJ 08901 USA

**Keywords:** ME-344, Colorectal cancer, OxPhos inhibition, VEGF, Metabolic synthetic lethality, Metabolomics

## Abstract

Antiangiogenic drugs may cause vascular normalization and correct hypoxia in tumors, shifting cells to mitochondrial respiration as the primary source of energy. In turn, the addition of an inhibitor of mitochondrial respiration to antiangiogenic therapy holds potential to induce synthetic lethality. This study evaluated the mitochondrial inhibitor ME-344 in combination with bevacizumab in patients with refractory metastatic colorectal cancer (mCRC). Patients were eligible if they had disease progression after standard therapies, adequate hematologic, hepatic and renal function, and no contraindications to bevacizumab. ME-344 was administered intravenously on days 1, 8 and 15 and bevacizumab on days 1 and 15 of 28-day cycles until disease progression or intolerance. The primary efficacy endpoint was progression-free survival (PFS) at week 16. In the 23 patients enrolled, the median age was 58 years, median number of prior lines of therapy was 4, and median interval from last therapy was 3 months. The most common adverse events (all grades/grade ≥ 3) were fatigue (48%/13%), abdominal pain (35%/4%), diarrhea (30%/4%) and constipation (30%/0%). No patient had an objective response; 9 patients (39%) achieved stable disease. The 16-week PFS was 30.6% (95% confidence interval [CI]: 12.2–51.3), the median PFS was 1.9 months (95% CI: 1.6–4.7), and the median overall survival was 6.7 months (95% CI: 3.4-not reached). ME-344 plus bevacizumab was well tolerated. Disease control was limited in this heavily pretreated patient population. Additional investigations in earlier lines are indicated, and extended-release ME-344 formulations may provide longer drug exposure to maximize benefit. (Trial registration number ClinicalTrials.gov NCT05824559. Registration date 22 March 2022).

**Supplementary Information:**

The online version contains supplementary material available at 10.1007/s10637-024-01489-1.

## Introduction

Significant progress has been made in the treatment of metastatic colorectal cancer (mCRC), particularly in the first- and second-line setting. Current standards include combination chemotherapy with the vascular endothelial growth factor (VEGF) inhibitor bevacizumab or, for *RAS* wild-type patients, an epidermal growth factor receptor (EGFR) inhibitor. Combinations targeting *BRAF*, immune checkpoint inhibitors, and HER-2 targeting therapies have further transformed treatment [1, 2]. However, resistance inevitably develops. Following exposure to a fluoropyrimidine, oxaliplatin, irinotecan, and applicable targeted agents, several agents have proven benefit. This includes the oral antiangiogenics regorafenib and fruquintinib, and the combination of trifluridine and tipiracil (TAS-102), with or without bevacizumab [3–7]. Unfortunately, the median progression-free survival (PFS) is only 2–3 months with single agent therapy, at best nearing 6 months with TAS-102 plus bevacizumab. These agents highlight the proven benefit of continuing to target the VEGF pathway in relapsed disease. Even so, the limited efficacy and adverse event profiles leave muted enthusiasm for these agents. As the median survival remains less than one year in refractory mCRC, there is clear need for alternate treatment options with different mechanisms of action.

ME-344 is an investigational synthetic small molecule mitochondrial inhibitor based on the isoflavone ring structure. It inhibits the oxidative phosphorylation pathway, which is involved in the production of adenosine triphosphate in the mitochondria [8, 9]. Preclinical screening studies have shown that ME-344 has broad antiproliferative activity against a panel of human cancer cells representative of most major organ systems. In a phase 1 study in solid tumors, the recommended phase 2 dose for further development was 10 mg/kg administered intravenously (IV) once weekly for 3 weeks in a 28-day cycle, with peripheral neuropathy representing the dose limiting toxicity at higher doses [10]. A subsequent phase 1b study confirmed that ME-344 at 10 mg/kg was well tolerated when administered in combination with topotecan in patients with ovarian and small cell lung cancer [11].

It has been shown that antiangiogenics can correct hypoxia and downregulate aerobic glycolysis in tumor cells [12, 13]. Tumors eventually adapt and show metabolic plasticity, switching to mitochondrial metabolism as the primary source of energy. Because of this adaptation, mitochondrial metabolism becomes essential for tumor survival. Therefore, combining an antimitochondrial agent with an antiangiogenic may result in metabolic synthetic lethality and improve disease control [14]. A randomized window of opportunity study in patients with early HER2-negative breast cancer has demonstrated that 3 doses of ME-344 in combination with a single dose of bevacizumab significantly decreased the proliferation biomarker Ki67 in tumors compared to bevacizumab alone, and the effect was more prominent in patients who had vascular normalization with bevacizumab as documented by 2[18 F]fluoro-2-deoxy-D-glucose (FDG)-PET [15]. This ME-344 mitochondrial on-target effect was verified by a decrease in succinate dehydrogenase and hypoxia inducible factor 1 subunit alpha (HIF1α) staining.

To test this hypothesis in a clinical setting where VEGF inhibitors are commonly used, we conducted this single arm phase 1b study in patients with refractory mCRC to evaluate the safety and efficacy of the combination of ME-344 and bevacizumab.

## Patients and methods

### Study conduct

The study was conducted by member institutions of the Academic Gastrointestinal Cancer Consortium (AGICC), funded by MEI Pharma, and registered on ClinicalTrials.gov under NCT05824559 and first posted on 22 March 2023. Each institutional Independent Review Board approved conduct of the study for the institution.

### Eligibility citeria

Patients at least 18 years of age were eligible if they had metastatic adenocarcinoma of the colon or rectum and measurable disease by RECIST v1.1. Patients must have had disease progression or intolerability to prior standard approved therapies, including fluoropyrimidine-, oxaliplatin-, and irinotecan-based chemotherapies, and cetuximab/panitumumab (for RAS wild-type tumors) in the advanced or metastatic setting. Patients with microsatellite instability-high/deficient mismatch repair (MSI-H/dMMR) CRC should have failed or demonstrated intolerance to prior programmed death receptor 1 (PD-1) blocking antibody. Patients with a *BRAF V600E* mutation should have failed or demonstrated intolerance to BRAF-targeted therapy. Prior treatment with an antiangiogenic agent was not required.

Other eligibility criteria included ECOG performance status 0 or 1, adequate bone marrow (hemoglobin ≥ 8.0 g/dL, platelet count > 100,000/mm^3^, neutrophil count > 1500/mm^3^), hepatic (total bilirubin ≤ 1.5 x the upper limit of normal (ULN), alanine aminotransferase and aspartate aminotransferase ≤ 2.5 x ULN or ≤ 5 × ULN for subjects with liver metastases), and renal (creatinine clearance ≥ 60 mL/min, urine protein < 2+) function. Key exclusion criteria included untreated or symptomatic central nervous system disease, contraindication to bevacizumab therapy, and uncontrolled hepatitis B, hepatitis C, or HIV infection.

### Investigational therapy and study assessments

ME-344 at 10 mg/kg was administered by 60-minute infusions on days 1, 8 and 15 of a 28-day cycles and bevacizumab at 5 mg/kg on days 1 and 15 of the cycle. Treatment was continued until disease progression based on Response Evaluation Criteria in Solid Tumors (RECIST) v1.1, symptomatic deterioration, intolerance, or withdrawal of consent. Tumor imaging, preferably by computerized tomography (CT) scan, was obtained at baseline, every 2 months for 6 months, every 3 months for 6 months and every 4 months thereafter.

### Pharmacokinetics

Serial blood samples for ME-344 pharmacokinetic analyses were collected 5 min after the end of ME-344 infusion and at 1, 2, 3, 6 and 24 h after the completion of infusion on days 1 and 15 of cycle 1, and ME-344 plasma concentrations were determined using validated analytical methods. ME-344 pharmacokinetic parameters were estimated using actual sampling times by non-compartmental analysis [16].

### Metabolomic sub-study

In one center, fasting plasma samples were collected for analysis of metabolites. Samples were collected on cycle 1 day 1 pre-dosing and 6 h post-dosing with ME-344, as well as pre-dosing on cycle 1 day 15 and day 1 of cycles 2 and 3. Samples were stored frozen until isolation of metabolites following the method described elsewhere [17]. Identification of metabolites was determined by untargeted LC-MS/MS using both negative and positive modes and extracted in EI-MAVEN software. MetaboAnalyst (RRID: SCR_015539) was utilized to determine enrichment and pathway analysis of the set of metabolites that changed by ≥ 20% between the samples collected on cycle 1 day 1 before and 6 h after ME-344 administration. GraphPad Prism was used for data visualization.

### Statistical plan

The study was designed to enroll patients in 2 sequential cohorts of approximately 20 patients each, with the second cohort opening to enrollment if the 16-week PFS rate in the first cohort exceeded 20%. The primary efficacy endpoint was PFS rate at week 16. Other efficacy endpoints included overall PFS, objective response rate using RECIST V1.1, and overall survival. With a sample size of 20 and 40 efficacy evaluable subjects, assuming the true PFS rate at 16 weeks was 30%, there would be an 89% and 94% probability, respectively, to observe a PFS rate ≥ 20% based on binomial distribution. The intent-to-treat (ITT) population was defined as all enrolled subjects who received at least 1 dose of ME-344, and the efficacy evaluable (EE) population was defined as all subjects who received at least 1 dose of ME-344 and had at least 1 adequate post baseline efficacy assessment. Time to event endpoints were analyzed using the Kaplan-Meier method. Proportions were reported with 2-sided exact 95% confidence interval (CI) based on the Clopper-Pearson method.

## Results

### Patient population

Between August and November 2023, a total of 23 patients were enrolled in Cohort 1. Median age was 58 years (range, 43–83), 52% of patients were female, and 78% were white. Site of the primary tumor was left colon in 10 (44%) patients, transverse/right colon in 6 (26%), and rectum in 7 (30%). The median number of prior lines of therapy, including adjuvant or neoadjuvant therapy where applicable, was 4 (range, 1–7). Details on demographics, disease characteristics, and prior therapies are shown in Table 1.

**Table 1:** Patient and disease characteristics

Characteristics	*N* = 23
Age, years Median (range)	58 (43, 83)
Sex, n (%) Female / Male	12 (52) / 11 (48)
Race, n (%) White Black Asian	18 (78) 2 (9) 3 (13)
Baseline ECOG performance status, n (%) 0 1 2	13 (57) 9 (39) 1 (4)
Primary tumor site, n (%) Left colon Rectum Right/ transverse colon	10 (44%) 7 (30%) 6 (26%)
Molecular mutations, n (%)* KRAS NRAS BRAF PIK3CA	(*N* = 17) 10 (59) 4 (24) 2 (12) 3 (18)
Time from last therapy to enrollment, months Median (range)	3 (0.5–27)
Prior systemic therapies Number prior therapies, median (range) Patients with ≥ 3 prior therapies, n (%)	4 (1–7) 19 (83%)
Type of prior therapies, n (%) Chemotherapy, n (%) Bevacizumab, n (%) Trifluridine/tipiracil Investigational agent Oral multikinase antiangiogenic EGFR inhibitor Immune checkpoint inhibitor	23 (100%) 23 (100%) 9 (39%) 7 (30%) 6 (26%) 5 (22%) 5 (22%)

ECOG: Eastern Cooperative Oncology Group, EGFR: Epidermal growth factor receptor

* Information not available for 6 patients

At final analysis, all patients have discontinued treatment, 16 (70%) due to disease progression, 2 (9%) due to investigator decision (1 for increased CEA and bilirubin and 1 for intolerance), 2 (9%) due to withdrawal of consent (1 for increased bilirubin and 1 transitioned to hospice care), 2 (9%) due to an adverse event (sepsis and fatigue), and 1 (4%) who was still on therapy at 7.8 months with stable disease when treatment was discontinued after the sponsor’s decision to close the study.

### Safety

Median duration of ME-344 therapy was 1.4 months (range, 0.3–7.8), and the median number of ME-344 infusions was 6 (range, 2–24). Adverse events reported in ≥ 10% of patients are shown in Table 2. The most common adverse events (all grades/grade ≥ 3) were fatigue (48%/13%), abdominal pain (35%/4%), diarrhea (30%/4%), and constipation (30%/0%). Peripheral sensory neuropathy was reported in 4 (17%) patients, 3 with grade 1 and 1 with grade 2 events. Except for 1 patient with reversible grade 4 thrombocytopenia, there were no other grade ≥ 3 hematologic toxicities. A serious adverse event was reported in 11 (48%) patients, and those reported in more than 1 patient were dehydration (2, 9%), pulmonary embolism (2, 9%), and sepsis (2, 9%). There was one death due to an adverse event, a 78-year-old patient who experienced clinical deterioration in cycle 1 and was referred to an assisted living facility where she fell, was hospitalized, developed sepsis, and died 1 month after last dose of study drug.

**Table 2:** Adverse events reported in ≥ 10% of patients

	Number (%) of Patients *N* = 23					
	Grade 1	Grade 2	Grade 3	Grade 4	Grade 5	All Grades
Fatigue	5 (21.7)	3 (13.0)	3 (13.0)	0	0	11 (47.8)
Abdominal pain	4 (17.4)	3 (13.0)	1 (4.3)	0	0	8 (34.8)
Diarrhea	2 (8.7)	4 (17.4)	1 (4.3)	0	0	7 (30.4)
Constipation	5 (21.7)	2 (8.7)	0	0	0	7 (30.4)
Nausea	2 (8.7)	2 (8.7)	1 (4.3)	0	0	5 (21.7)
Vomiting	4 (17.4)	1 (4.3)	0	0	0	5 (21.7)
Hyponatremia	2 (8.7)	2 (8.7)	0	0	0	4 (17.4)
Peripheral sensory neuropathy	3 (13.0)	1 (4.3)	0	0	0	4 (17.4)
Aspartate aminotransferase increased	3 (13.0)	0	0	0	0	3 (13.0)
Alkaline phosphatase increased	1 (4.3)0	2 (8.7)	0	0	0	3 (13.0)
Bilirubin increased	1 (4.3)	0	2 (8.7)	0	0	3 (13.0)
Decreased appetite	0	1 (4.3)	2 (8.7)	0	0	3 (13.0)
Dehydration	1 (4.3)	0	2 (8.7)	0	0	3 (13.0)
Headache	3 (13.0)	0	0	0	0	3 (13.0)
Hypertension	0	1 (4.3)	2 (8.7)	0	0	3 (13.0)
Leukocytosis	0	0	3 (13.0)	0	0	3 (13.0)
Non-cardiac chest pain	3 (13.0)	0	0	0	0	3 (13.0)

### Efficacy

**Fig. 1:**
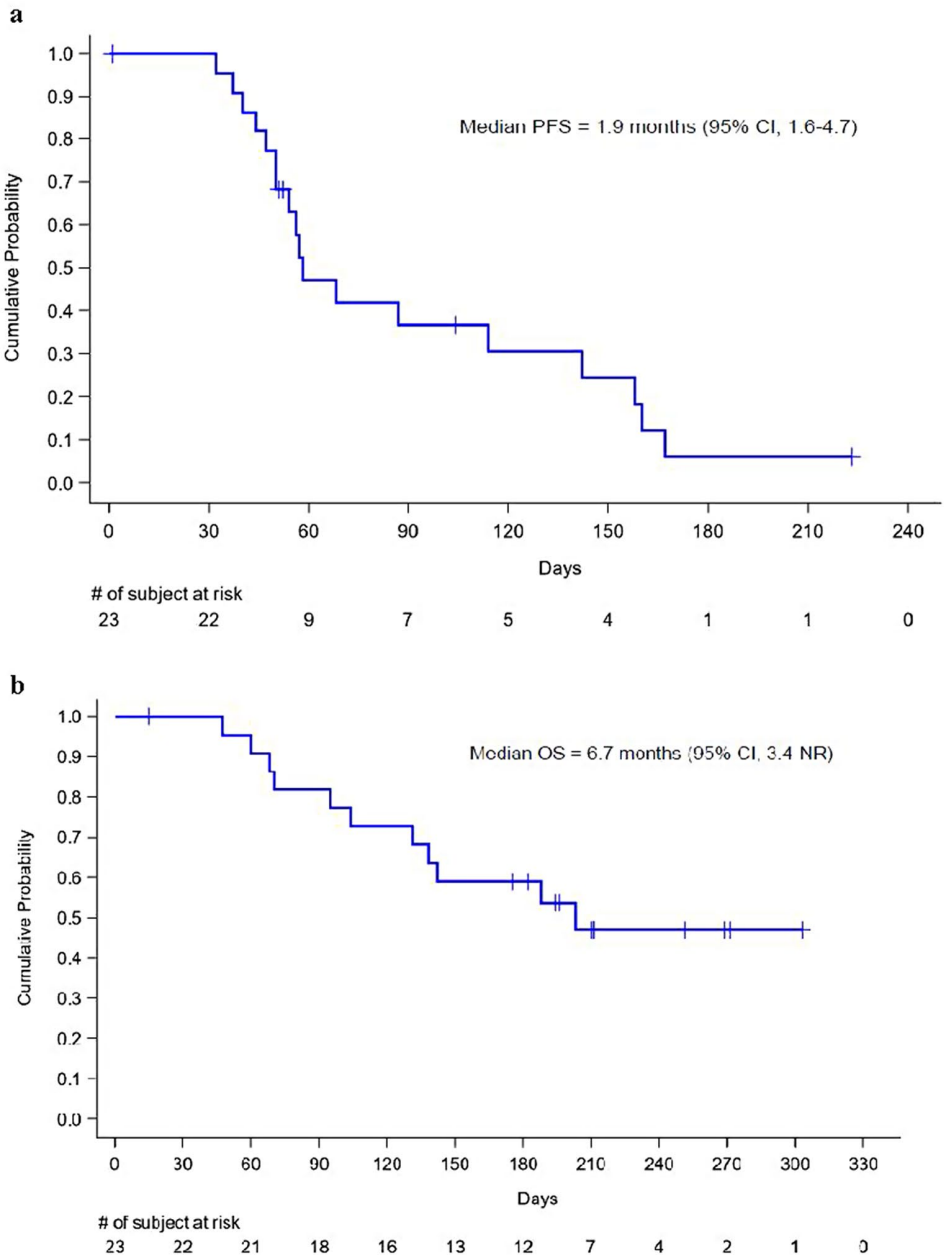
Progression-free survival (**a**) and overall survival (**b)** PFS progression-free survival, OS overall survival, CI confidence interval, NR not reached

Post baseline disease assessment was not available in 3 patients who discontinued study drug in the first cycle, 1 due to an adverse event and 2 due to withdrawal of consent. No objective responses were observed in the 20 patients who were evaluated for disease status radiographically, and 9 (39%) patients had stable disease. Five of 23 patients (22% [95% CI: 7.5–43.7]) of the intent-to-treat population, corresponding to 5 of 20 patients (25% [95% CI: 8.7–49.1]] of the efficacy evaluable population, did not have evidence of disease progression at the week 16 assessment, exceeding the 20% predefined threshold to open cohort 2. However, patient enrollment was closed by the sponsor to reprioritize the development of a new formulation with longer plasma exposure. The estimated 16-week PFS was 30.6% (95% CI, 12.2–51.3), median PFS was 1.9 months (95% CI, 1.6–4.7) and median overall survival was 6.7 months (95% CI, 3.4-not reached), with the Kaplan Meier plots shown in Fig. 1.

### Pharmacokinetics

**Fig. 2:**
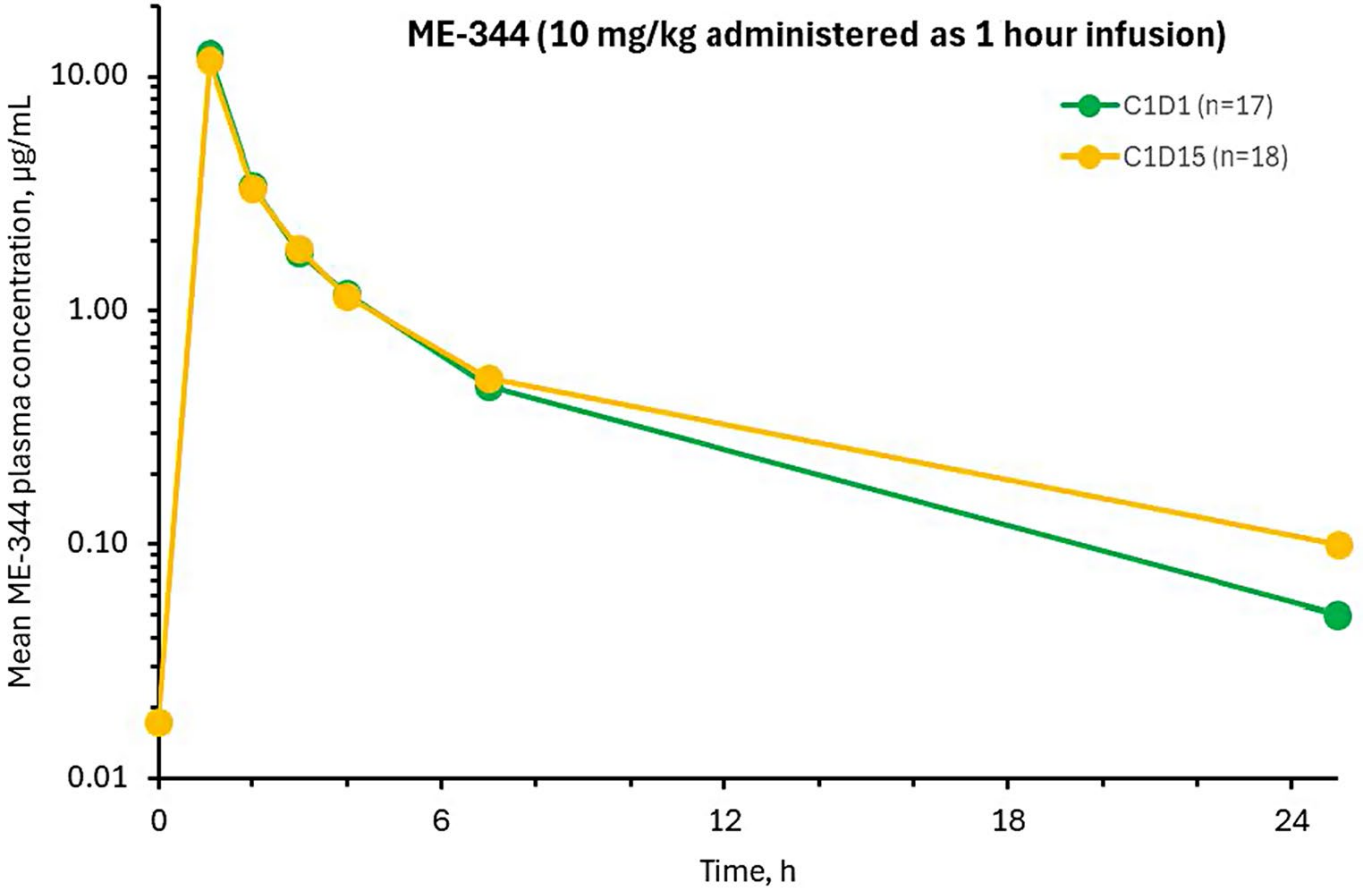
Mean ME-344 plasma concentration-time profile on cycle 1 days 1 and 15. C1D1 cycle 1 day 1, C1D15 cycle 1 day 15

Maximum plasma ME-344 concentrations (C_max_) were observed immediately after the end of infusion and plasma concentrations declined in a multiexponential manner with an average terminal phase half-life (t_1/2_) of approximately 4.4 h and 5.1 h on day 1 and day 15, respectively (Fig. 2). ME-344 pre-dose plasma concentrations were negligible on day 15, indicating near complete elimination of previously administered doses of ME-344 from systemic circulation. Mean accumulation index values for C_max_, area-under-the-curve (AUC) variables AUC_last_ and AUC_inf_ were 1.14, 1.19 and 1.28, respectively, on day 15 indicating minimal to no accumulation after once weekly administration. Variability in ME-344 pharmacokinetic parameters, expressed as percent coefficient of variation, was moderate (Supplemental Table 1).

### Metabolomics analysis

**Fig. 3:**
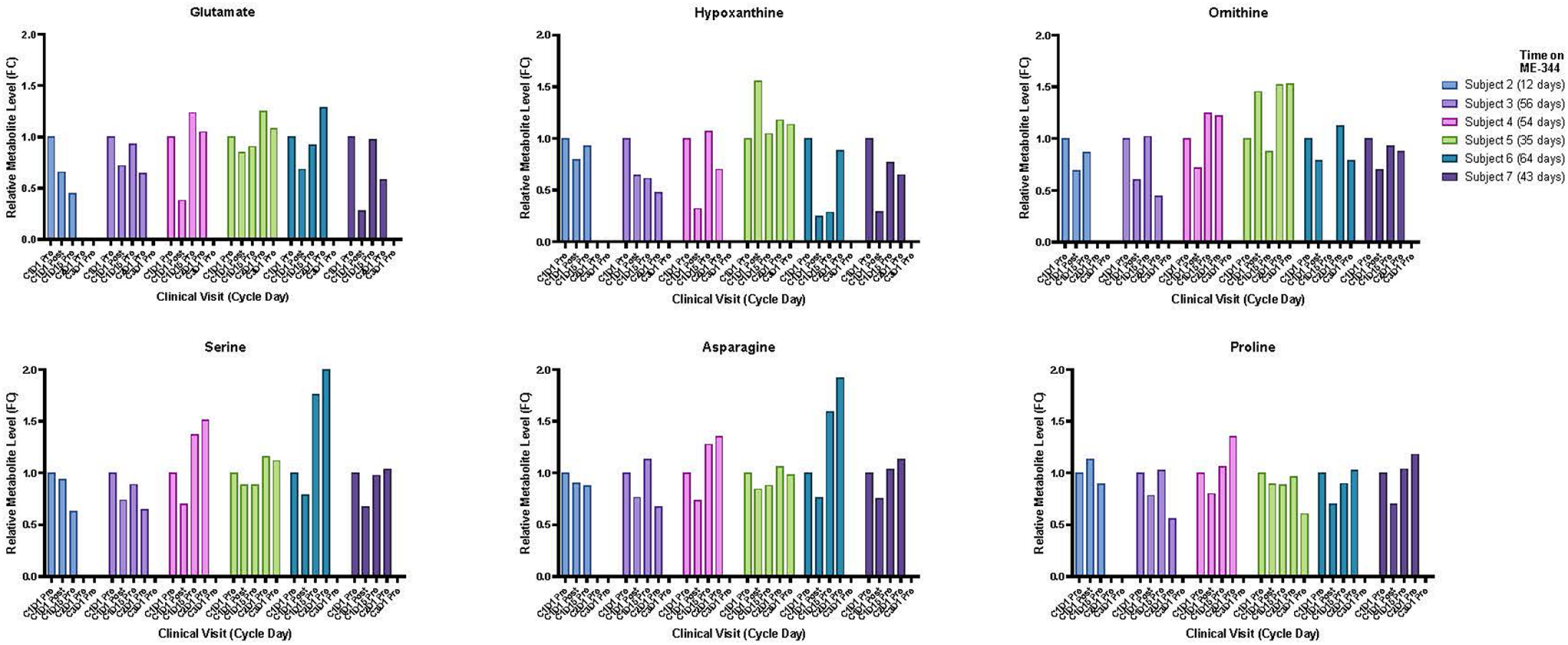
Metabolites with ≥ 20% change from baseline. LC-MS/MS results for selected metabolites expressed as fold change (FC) from baseline (cycle 1 day 1, pre-dose). Missing data is due to lack of samples at all timepoints. The top row of graphs are from data collected in positive mode and the bottom row from negative mode

Fasting plasma samples were collected from 6 patients over time during cycles 1 to 3. Untargeted metabolomics analysis was performed using LC/MS-MS in both negative and positive modes that identified 61 and 52 metabolites, respectively (Supplemental Fig. 1A and B). To identify metabolites that acutely responded to ME-344, we focused on metabolites that showed a ≥ 20% change in ≥ 4 patients between samples collected at baseline and 6 h after ME-344 administration. We observed ≥ 20% decrease in 14 metabolites in negative mode analysis and 10 metabolites in positive mode analysis. In total, there were 18 unique metabolites that dynamically changed in at least 4 patients in response to the initial ME-344 administration (Fig. 3 and Supplemental Fig. 2). Amino acids and amino acid derivatives represented 78% of the 18 metabolites acutely modulated by ME-344. Purine nucleotides comprised 17% of the 18 metabolites. Most metabolites had returned to baseline levels by day 15 when the pre-dose sample was collected, which was a week after the previous dose of ME-344. Pathway and enrichment analyses of the 18 changed metabolites suggested that metabolism of amino acids and purines were significantly represented (Supplemental Table 2). Notably, glutamate, serine, ornithine, proline, asparagine, and hypoxanthine, which have been associated with survival or progression of CRC cells, were acutely decreased, particularly in subjects who remained on ME-344 therapy the longest [18] (Fig. 3).

## Discussion

This single-arm, multi-center phase Ib study sought to evaluate the efficacy of ME-344 combined with bevacizumab in patients with refractory mCRC. In total, 23 patients were enrolled rapidly over a period of 4 months, indicating an unmet need for patients with refractory disease. Treatment was found to be safe, without new safety signals. Unfortunately, the clinical activity was limited, achieving a median PFS of 1.9 months, estimated 16-week PFS rate of 30.6%, and a median overall survival of 6.7 months.

Angiogenesis has a crucial role in tumor growth and metastases, and angiogenic inhibiting drugs have become a major therapeutic approach in several common epithelial malignancies such as colorectal, lung, kidney and liver cancers [19, 20]. Two main types of antiangiogenics have been developed, monoclonal antibodies aimed VEGF or VEGF receptors and the oral small molecule multi-tyrosine kinase inhibitors (TKIs) involved in normal cellular functions and oncogenesis [21]. However, single agent activity is modest, and development of resistance is common, with several mechanisms involved [22]. Various strategies have been studied to enhance antiangiogenic therapy, including combination with chemotherapy, targeted therapy, or immune-checkpoint inhibitors, the latter based on the known immunosuppressive effects of VEGF, which can be reversed with VEGF inhibition [23].

Another approach attempts to capitalize on hypoxia correction following antiangiogenic therapy leading to downregulation of glycolysis and switch to long-term reliance on mitochondrial respiration for tumor survival. In vivo, agents like ME-344 can induce synergistic tumor control when combined with antiangiogenics, leading to metabolic synthetic lethality, as shown in combination with TKIs in the PyMT breast cancer and Lewis lung carcinoma models [14]. This effect was subsequently documented in a randomized phase 0/1 window of opportunity study in 42 patients with newly diagnosed HER2-negative breast cancer, where patients were initially treated with a single dose of bevacizumab with pre- and post-dose FDG-PET, followed by randomization to ME-344 + bevacizumab or placebo. Those patients whose tumors had evidence of vascular normalization by FDG-PET (and confirmed by confocal microscopy and HIF1α staining) had a greater decrease in Ki67 [15].

We conducted this study in patients with refractory mCRC given the poor outcomes with standard of care therapy and the established use of antiangiogenics [24]. Because we had no clinical data on ME-344 in combination with bevacizumab in mCRC, this study was designed as having two stages, with the second stage opening to enrollment if at least 20% of patients enrolled in the first stage were progression free at week 16, doubling the rate observed in the phase 3 study of regorafenib in refractory mCRC [3]. While the observed 16-week PFS was 25% in cohort 1, meeting the predefined protocol criteria to open cohort 2, further enrollment to the study was halted by the sponsor to instead prioritize the development of an extended-release formulation which may provide longer exposure to ME-344 and possibly better synergy with antiangiogenics. ME-344 plasma concentrations in this study were generally comparable to exposures previously reported with single agent ME-344, as well as ME-344 in combination with topotecan, with a mean half-life of approximately 6 h [10, 11].

Patients enrolled in the study were heavily pretreated with a median of 4 prior lines of therapy, including bevacizumab in all. In this population, treatment was well tolerated with no drug-related adverse events resulting in treatment discontinuation. The estimated median PFS of 1.9 months and median overall survival of 6.7 months are comparable to that reported with either TAS-102 or regorafenib alone. Of note, patients enrolled in the regorafenib phase 3 study, were all TKI-naïve, whereas 26% of patients enrolled in this study had disease that progressed after TKI therapy [3]. There are no published data with bevacizumab monotherapy in this setting. Thus, it is not possible to determine how the clinical activity might differ from bevacizumab alone. The bevacizumab dose of 5 mg/kg every 14 days was selected based on the prior experience in the breast cancer study and the same as administered with the FOLFOX and FOLFIRI regimens, but lower than 10 mg/kg dose that was evaluated in the phase 3 Study E3200 comparing FOLFOX4 with or without bevacizumab or bevacizumab alone [25]. It is conceivable that a higher bevacizumab dose may have achieved greater synergy with ME-344 leading to a higher response rate. Further, as seen in the phase 0/1 breast cancer study, pre-selection of patients with post-bevacizumab vascular normalization, as judged by FDG-PET, may have enriched for a patient population that benefitted from this strategy.

The untargeted serum metabolomics study revealed considerable variation in baseline levels of metabolites across subjects; however, there was an acute decrease of ≥ 20% in several purine nucleotides and amino acids, including glutamate, serine, ornithine, proline, asparagine, and hypoxanthine. All these metabolites have been associated with proliferation of CRC cell lines or tumors [18]. The 20% cutoff was chosen empirically for this exploratory study, and it is possible that a higher cutoff may have been more clearly associated with better outcomes. The goal of this sub-study was to identify potential metabolic biomarkers of response to ME-344 that could serve as the basis for a targeted panel of metabolites to interrogate in future studies, which could be comprised of a panel of 6 to 18 of the identified metabolites. As noted, the observed metabolites largely returned to near baseline levels prior to dosing on day 15. Thus, while this analysis suggests modulation of metabolic pathways relevant to mitochondria, it also raises concern that exposure may be suboptimal, with a half-life of 6 h and dosing being just weekly.

In conclusion, this study showed that ME-344 in combination with bevacizumab was well tolerated, with no increased toxicity compared to what was reported with each agent alone. Some activity was seen with a degree of disease stabilization in heavily pre-treated patients, not expected with bevacizumab alone. Further investigation can be considered, preferably using an ME-344 extended-release formulation that would increase exposure to study drug and potentially improve clinical benefit.

## Electronic Supplementary Material

Below is the link to the electronic supplementary material.

Supplementary Material 1

## Data Availability

No datasets were generated or analysed during the current study.
