# Supplementary material for: A Phase 1b study of the OxPhos inhibitor ME-344 with bevacizumab in refractory metastatic colorectal cancer
Source: Invest New Drugs. 2024 Dec 27;43(1):60–8. doi: 10.1007/s10637-024-01489-1 (PMC11868331; doi:10.1007/s10637-024-01489-1)
Supplement: Supplementary file 1 — Supplementary Material 1 [file 10637_2024_1489_MOESM1_ESM.docx]

**SUPPLEMENTAL MATERIAL**

**Supplemental Table 1: ME-344 pharmacokinetic parameters**

| **Parameter**  **Mean (%CV)** | **Cycle 1 Day 1**  **(n = 17)** | **Cycle 1 Day 15**  **(n = 18)** |
| --- | --- | --- |
| C_max_, µg/mL | 12.60 (42%) | 11.83 (47%) |
| T_max_, hours, median (range) | 1.08 [1.03 – 1.33] | 1.17 [1.03 – 2.23] |
| AUC_last_, µg×h/mL | 22.6 (34%) | 22.4 (35%) ^A^ |
| AUC_inf_, µg×h/mL | 23.0 (34%) | 23.7 (38%) ^A^ |
| Half-life, h | 4.4 (20%) | 5.1 (38%) ^A^ |
| Accumulation index C_max_ |  | 1.14 (44%) ^B^ |
| Accumulation index AUC_last_ |  | 1.19 (24%) ^C^ |
| Accumulation index AUC_inf_ |  | 1.26 (32%) ^C^ |

^A^ n = 15; ^B^ n = 12; ^C^ n =10

%CV percent coefficient variation; C_max_ maximum plasma concentration, T_max_ time to maximum plasma concentration, AUC area-under-the-curve; h hour; mL milliliter.

**Supplemental Table 2: Pathway analysis**

| **Pathway** | **Raw p-value** |
| --- | --- |
| Arginine and proline metabolism | 0.002096 |
| Arginine biosynthesis | 0.004605 |
| Purine metabolism | 0.013861 |
| Glutathione metabolism | 0.018024 |
| Alanine, aspartate and glutamate metabolism | 0.018024 |
| Glyoxylate and dicarboxylate metabolism | 0.023254 |
| Nitrogen metabolism | 0.044922 |
| Valine, leucine and isoleucine biosynthesis | 0.05948 |
| Taurine and hypotaurine metabolism | 0.05948 |
| Biotin metabolism | 0.073835 |
| D-Amino acid metabolism | 0.10885 |
| Butanoate metabolism | 0.10885 |
| Histidine metabolism | 0.1157 |
| Pantothenate and CoA biosynthesis | 0.14264 |
| Lysine degradation | 0.20673 |
| Porphyrin metabolism | 0.21289 |
| Sphingolipid metabolism | 0.21901 |
| Cysteine and methionine metabolism | 0.22508 |
| Glycine, serine and threonine metabolism | 0.22508 |
| Valine, leucine and isoleucine degradation | 0.2664 |
| Tryptophan metabolism | 0.27214 |

**Supplemental Fig 1. Heat maps of metabolites collected in positive (A) and negative modes (B).**

**A.**

**B.**

LC-MS/MS ion count results for all metabolites detected in negative (A) and positive (B) modes. Missing data is due to lack of samples at all timepoints. A zero (0) is in the box if no metabolite was detected in the sample.

**Supplemental Fig 2. Additional metabolites with changes ≥20% from baseline.**

LC-MS/MS results for selected metabolites expressed as fold change (FC) from baseline (cycle 1 day 1, pre-dose). Missing data is due to lack of samples at all timep
